# When a patient suspected with juvenile idiopathic arthritis turns out to be diagnosed with an infectious disease – a review of Lyme arthritis in children

**DOI:** 10.1186/s12969-017-0166-0

**Published:** 2017-05-08

**Authors:** Krzysztof Orczyk, Joanna Świdrowska-Jaros, Elżbieta Smolewska

**Affiliations:** Department of Pediatric Rheumatology, Medical University of Lodz, Sporna 36/50, 91-738 Lodz, Poland

**Keywords:** Lyme arthritis, Lyme disease, Pathogenesis, Symptoms, Treatment

## Abstract

The Lyme arthritis is a common manifestation of infection with *Borrelia burgdorferi* spirochete. Despite its infectious background, the inflammation clinically and histopatologically resembles juvenile idiopathic arthritis. As it affects a considerable number of Lyme disease patients, it should be routinely considered in differential diagnosis. Development of arthritis is partially dependent on spirochetal factors, including the ribosomal spacer type and the sequence of outer surface protein C. Immunological background involves Th1-related response, but IL-17 provides an additional route of developing arthritis. Autoimmune mechanisms may lead to antibiotic-refractory arthritis. The current diagnostic standard is based on a 2-step testing: ELISA screening and immunoblot confirmation. Other suggested methods contain modified two-tier test with C6 ELISA instead of immunoblot. An initial 28-day course of oral antibiotics (doxycycline, cefuroxime axetil or amoxicillin) is a recommended treatment. Severe cases require further anti-inflammatory management. Precise investigation of new diagnostic and therapeutic approaches is advisable.

## Background

Lyme arthritis (LA) is a feature of late stage infection with the tick-borne spirochete, *Borrelia burgdorferi (B. burgdorferi)*. It usually occurs weeks to months after the initial tick bite and may be preceded by erythema migrans (EM) [1]. This erythematous patch with a characteristic central clearing forming a typical “bull’s eye lesion” is considered to be a pathognomonic manifestation of Lyme disease (LD) [2]. However, there is a growing body of evidence that the earlier stage of the infection frequently tends to remain asymptomatic and arthritis is becoming the presenting manifestation of the disease [3].

Steere et al. were the first to describe LD in Lyme, Connecticut, the United States, in patients initially suspected of juvenile idiopathic arthritis (JIA) [4]. Thorough diagnostic process in the aforementioned group revealed that reported episodes of arthritis were provoked by a novel infectious trigger [5]. To date, there are 20 identified genospecies of *B. burgdorferi sensu lato* [6]. Nine of them infect humans: *B. burgdorferi sensu stricto, B. garinii, B. afzelii, B. bavariensis, B. bissetii, B. kurtenbachii, B. lusitaniae, B. spielmanii, B. valaesiana* [7]. The highest arthrogenic potential is presented by *B. burgdorferi sensu stricto* [8]. It induced arthritis in 46% of infected patients in the United States observed by Cerar et al. [9]. Its immunogenicity plays a crucial role in differences in clinical course of spirochetal infection between the United States, where *B. burgdorferi sensu stricto* is the main etiological factor of LD [10], and Europe, which is dominated by two other genospecies: *B. garinii* and *B. afzelii* [11]. As reported by Cerar et al. who assessed clinical images of LD in Slovenia, patients infected with *B. garinii* and *B. afzelii* developed arthritis in 18 and 15%, respectively [9]. According to Kocbach et al., *B. garinii* infection frequently produces neurological symptoms and patients infected with *B. afzelii* regularly present with fatigue and myalgia [8].

### Epidemiology

LD usually affects people in northern hemisphere [12]. The highest incidence in Europe was reported in southern Sweden (464/100,000) [13]. Sykes et al. calculated average incidence rate in Western Europe as 22,05/100,000, what results in approximately 230,000 of cases in Western Europe per year [14]. In the United States, The Centers for Disease Control and Prevention (CDC) reported nearly 30,000 of cases of LD in 2008 [15], whereas a survey regarding LD testing performed by large commercial laboratories gave a result of estimated 300,000 of cases per year [16].

There is a tendency towards an upswing in incidence of LD in the last few years [17]. Incidence rate reported in Poland in 2014 was 36/100,000 and it was 52,9% higher than median from years 2008–2012 [18]. It is caused not only by better awareness of the disease and changes in human recreational behavior, but also by dispersion of reservoir hosts [19]. Colonization of urban areas in Poland by blackbirds [6] may provide a potential explanation, as one of the genospecies which dominate in Europe, *B. garinii*, most commonly affects birds [20].

There are two peaks of age in incidence of spirochetal infection. Most cases of LD are reported in age groups which spend more time outdoors near the natural reservoirs of ticks, specifically children between 5 and 9 years of age and adults >50 years [21]. Period between tick bite and its removal is considered to be a pivotal risk factor for infection [22], which is more likely to occur if a tick remains attached to the skin for more than first 24 h after bite [23]. Owning dogs has been also considered as a potential risk factor for LD, but Aenishaenslin et al. did not find significantly higher risk of tick bites in dog owners [24].

LA affects 3–15% of LD patients in Europe [25]. As for the United States, CDC reported LA in 33% of LD patients diagnosed in 2001 and 2002 [26]. LD caused 51% of pediatric knee arthritis (341 out of 673 patients) observed by Deanehan et al. [27] and 5,2% of pediatric hip arthritis (20 out of 385 patients) reported by Bachur et al. [28]. Therefore it is frequent enough to be included in differential diagnosis, which should consider other inflammatory arthritides, such as reactive arthritis and JIA (including psoriatic arthritis). Autoinflammatory arthritis may also develop within months after LD, presumably triggered by the spirochetal infection [3].

### Clinical features

**Fig. 1:**
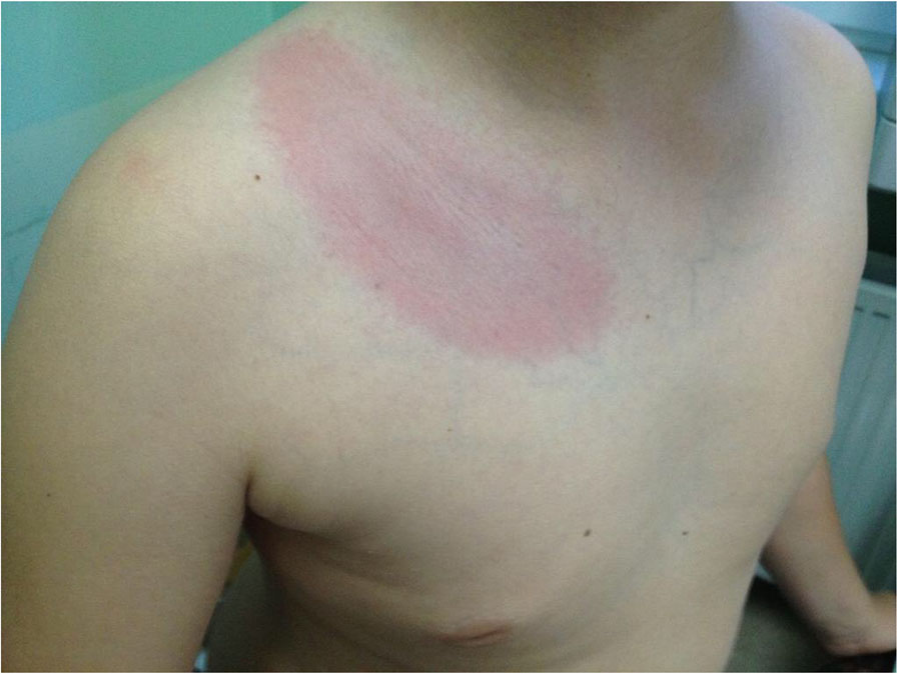
Erythema migrans in a 9-year-old boy after tick bite

EM (Fig. 1), a characteristic sign mentioned above as a common initial manifestation of *B. burgdorferi* infection, is frequently accompanied, or sometimes preceded, by systemic symptoms, in particular fatigue, fever, headache, stiff neck, myalgias, arthralgias, nausea and dysesthesia [29]. However, according to Glaude et al., the majority (72%) of patients observed in Canada had arthritis as their first symptom of LD. 76% of patients did not recall a tick bite, and only 18% of patients had previous EM [30].

In patients who developed EM, arthralgias appeared 1 day to 8 weeks after onset of rash in a study of 55 patients performed by Steere et al. [31]. When EM is absent, symptoms of LA may occur months or even years after initial tick bite [32]. Therefore, it can be manifested in every month of the year, not only within time of increased outdoor activity [33]. In accordance with aforementioned diversity of genospecies, LA observed in Europe (caused mainly by *B. garinii* and *B. afzelii)* tends to occur in shorter period after initial tick bite comparing to *B. burgdorferi sensu stricto* infections [9].

On clinical presentation, LA usually affects large synovial joints asymmetrically [34]. Recurrent swelling of the affected joint with a presence of moderate inflammatory effusion is a common clinical finding in children suspected of LA [35]. Although more acute presentation may mimic septic arthritis, pain of the affected joints reported in patients with LA is usually less severe [27]. All patients reported by Huppertz et al. experienced joint swelling or limitation of range of motion, but no pain [36]. Additionally, 38% of patients assessed by Thompson et al. were incapable of bearing weight on the affected limb [37].

**Fig. 2:**
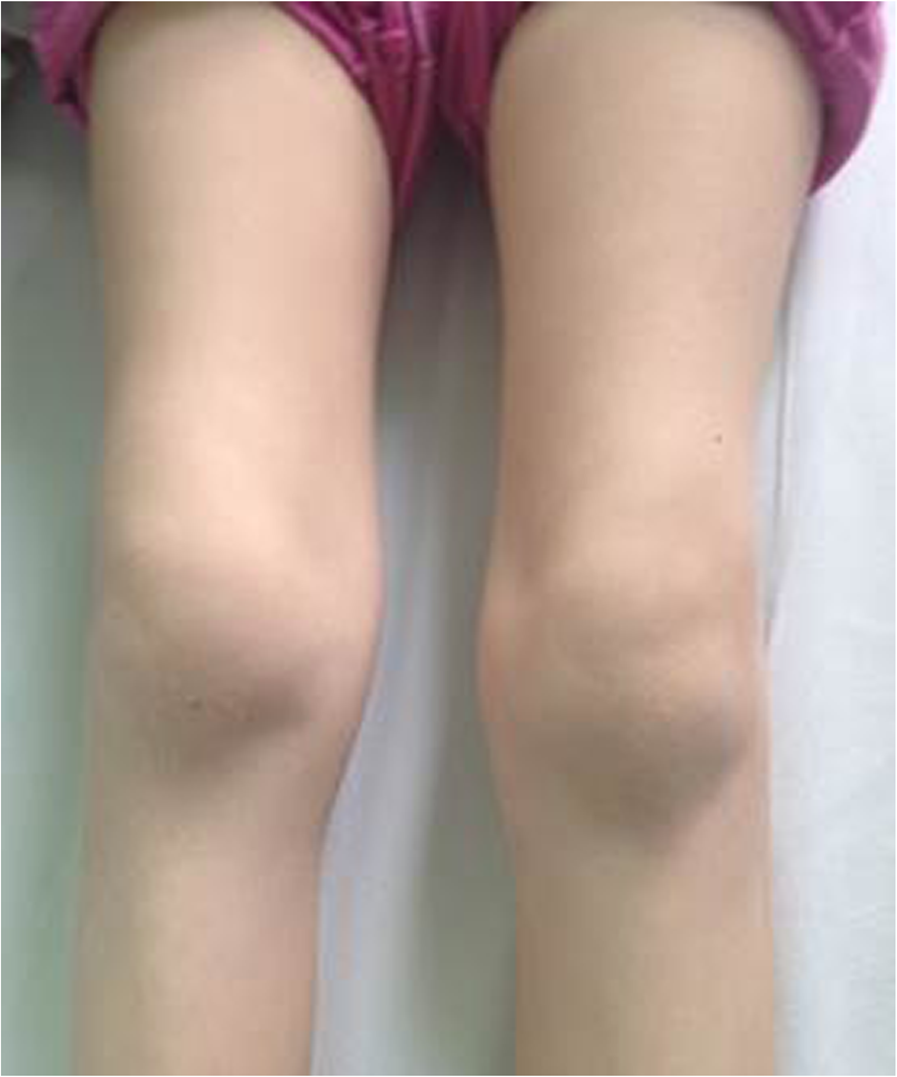
Bilateral knee arthritis in a serological-positive (*Borrelia burgdorferi*) 6-year-old girl

The knee is affected (Fig. 2) in over 90% of cases [38]. The ankle is found to be the second most commonly involved joint [38]. Correspondingly, the clinical image can resemble a mono- or oligoarticular JIA. Still, polyarticular involvement should not automatically exclude LA. Sá et al. reported a case of a 6-year-old girl with serological confirmation of spirochetal infection who developed swelling of the proximal inter-phalangeal joints of hands and wrist and both tibiotarsal joints within several months after initial tick bite and was finally diagnosed with polyarticular JIA [39].

LA that does not recover during first 3 months after initiation of recommended treatment regimens (described below) has been referred to as antibiotic-refractory LA [36, 40]. In a study published by Tory et al. 23% of pediatric patients had persistent synovitis after 8 weeks of oral antibiotic therapy or 4 weeks of intravenous therapy or both [41]. LA eventually resolves spontaneously, but this may take even 8 years in extreme cases [42].

### Spirochetal factors of inflammation

The *B. burgdorferi* genome has a unique structure. More than 40% of genetic material is located in linear and circular plasmids therefore spirochetes are able to rapidly adapt to new environmental conditions [43]. *B. burgdorferi* genotypes are classified on the basis of the 16S-23S ribosomal spacer type (RST) and the sequence of outer surface protein C (OspC) [9]. RST1 strains are more likely to cause disseminated *B. burgdorferi* infection, whereas RST2 and RST3 strains are less frequently detected peripherally. Moreover, RST1 isolates induce significantly greater interferon (IFN)-γ production by peripheral blood mononuclear cells comparing to RST3 strains [44]. Infection of C3H/HeJ mice with RST1 strains resulted in more severe arthritis and carditis than did infection with RST3 isolates in a study performed by Wang et al. [45].

Spirochetal antigen OspC is a factor crucial for the invasion of a host [46]. OspC facilitates bacterial transmission with tick saliva during a bite by forming a complex with a tick salivary protein, Salp15 [47]. OspC is highly immunogenic and is one of the primary inductors of host immune response [48]. Moreover, it interacts with plasminogen which is likely to enable spirochetal dissemination to peripheral tissues [46].

Through its surface molecules, *B. burgdorferi* is able to bind host proteins and, in that way, hide from the host immune system. As reviewed by Nardelli et al., it can bind to fibronectin, type I collagen, glycosaminoglycans, proteoglycans and integrins [49].

*B. burgdorferi* has also been shown to rapidly inhibit human complement by binding host complement regulators (such as FH, FHR-1, C4Bp, C1r, C7, C8, C9, TCC) through a family of surface-exposed molecules (CspA, CspZ, ErpP, ErpC, ErpA, p43, BBK32, BGA66, BGA71 and CD59-like protein) [50].

Additionally, *B. burgdorferi* produces protease with aggrecanase activity, which is postulated by Russell et al. to be involved in direct mechanism of tissue damage in LA [51].

There is also evidence of roles for several constituents of tick saliva. Prostaglandin E2 inhibits the production of the T helper (Th)1-associated cytokines interleukin (IL)-12 and tumor necrosis factor (TNF)-α by dendritic cells in an infection site [52]. B-cell inhibitory protein and sialostatin L are also involved in local suppression of immune response [53, 54].

### Immunological background

**Fig. 3:**
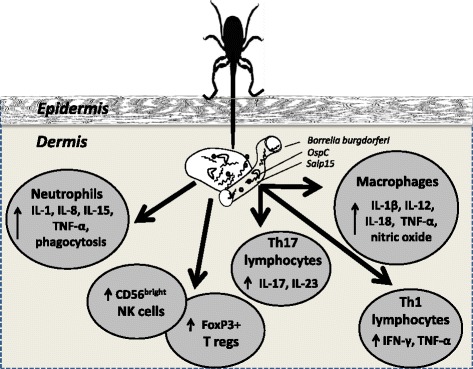
Set of changes in cytokine profile and concentrations of cells in the pathogenesis of LA; abbreviations explained in the text

Presence of spirochetes at synovial sites triggers development of joint inflammation (Fig. 3) characterized by effusion dominated with neutrophils. As they bind spirochetal lipoprotein to Toll-like receptor 2 [55], neutrophils produce proinflammatory cytokines: IL-1, TNF-α, IL-8 and IL-15 [49]. They can also eliminate spirochetes through phagocytosis [56].

Additionally, spirochetal stimulation of macrophages results in release of proinflammatory mediators typical for M1 macrophages, namely: TNF-α, IL-1β, IL-12, IL-18 and nitric oxide [57].

Subsequently, development of joint inflammation involves Th1 lymphocytes. Activated T lymphocytes from LD patients produce IFN-γ following *ex vivo* stimulation with *B. burgdorferi* constituents [58]. In addition, IFN-γ and IFN-responsive genes are expressed in the joints of *B. burgdorferi*-infected mice [59, 60]. Surprisingly, the levels of IFN-γ did not correlate with the severity of other symptoms in a study performed by Callister et al. [61].

Zeidner et al. found that treating mice with the Th1-associated cytokines TNF-α and IFN-γ during tick feeding provided a 95 and 55% protection rate against transmission, respectively [62].

Th1-related inflammation is not the only possible immunological route of developing LA. *B. burgdorferi* has been shown to be a strong inducer of IL-10 which inhibits Th1 activity and may contribute to the immune tolerance of the microbe [63].

Several studies reviewed by Kuo et al. showed that arthritis observed in IFN-γ-deficient mice was dependent on IL-17 [64]. Infante-Duarte et al. reported compatible results in humans [65]. However, Bachmann et al. did not observe production of IL-17 by *B. burgdorferi*-stimulated human peripheral blood mononuclear cells [66].

Production of IL-17, as well as another Th17-supporting cytokine, IL-23, is induced by borrelial neutrophil activating protein A (NapA) [67]. There is mounting evidence that levels of these cytokines tend to be elevated in patients with severe, persisting symptoms, namely in antibiotic-refractory LA [67, 68].

Patients with LA have also high frequencies of CD56bright natural killer (NK) cells in synovial fluid [69]. It has been suggested that CD56bright NK cells may either exacerbate or regulate immune responses [70]. Presence of CD56bright NK cells producing IFN-γ in synovial fluid suggests that these cells contribute to elimination of spirochetes [52]. Barthold et al. reported less severe LA in mice genetically deficient in granulocytes and NK cells [71].

FoxP3+ regulatory T cells in synovial fluid also play a role in LA [72]. Low frequencies of FoxP3+ regulatory T cells in synovial fluid lead to slower arthritis resolution [73, 74].

The progression into antibiotic-refractory LA is presumably independent of persistent spirochetal infection. Underlying autoimmune background has been postulated by Steere et al. [75]. There are seven human leukocyte antigen-D-related (HLA-DR) alleles identified, including the DRB1*0101, 0401 and 1501 alleles, that are predominant in patients with antibiotic-refractory patients [76]. Moreover, Arvikar et al. listed 4 Lyme-associated autoantigens that are more frequent in the aforementioned group [77]. They include: endothelial cell growth factor (ECGF), apolipoprotein B-100 (apoB-100), matrix metalloproteinase-10 (MMP-10) and annexin-A2. Of these, only the response to annexin-A2 is not LA-specific, as it is elevated in both LA and RA [78].

## Diagnostics

The current diagnostic standard of LA requires both clinical and laboratory criteria [79]. Laboratory testing relies predominantly on serology. Overall, a two-step approach is recommended: initial screening with an enzyme-linked immunosorbent assay (ELISA) followed by a supplemental Western immunoblot test (WB) in case the first step is positive [80]. In the United States, current criteria for a positive WB established by CDC require the presence of 2 (of 3) specified bands on the IgM WB or 5 (of 10) specified bands on the IgG WB [81]. There are also several other types of immunoblotting assays (including the EUROIMMUN Anti-Borrelia EUROLINE-WB which is frequently utilized in Europe) with variation in antigen composition and modified interpretation criteria which are provided by the producers [82].

Patients with signs and symptoms ≤30 days may be considered positive based on either IgM or IgG, whereas those with signs and symptoms >30 days, that is the majority of patients with LA, require IgG positivity [83]. Serology results may be falsely positive due to cross-reactivity, especially in patients with infectious mononucleosis [84] or in the presence of rheumatoid factor (RF) [7].

The diagnostic value of testing LA patients for IgM is questionable. A positive IgM WB may be falsely positive, particularly if a patient has manifested symptoms for more than 1 month [85]. Sensitivity of IgM testing in LA patients assessed by Leeflang et al. was insufficient (0.392) to be considered reliable, whereas IgG testing was sensitive enough (0.941) to be informative [86]. Specificity of IgM and IgG was comparable: 0.951 and 0.969, respectively.

Serology results are inadequate to monitor the treatment of LA [7]. It may be expected that some individuals with LA will be found to have positive IgM WB months to years later, due to the persistence of OspC-specific antibody response [87]. Polymerase chain reaction (PCR) on synovial fluid performed no sooner than 2 months after treatment is postulated to serve as a marker of eradication of spirochetes [87]. However, it is not yet properly standardized to use routinely in the diagnostic process [88].

There are several limitations of WB which affect the results of the standard two-tier test. As reviewed by Branda et al., it is insensitive in early phase of LD due to slow development of the humoral immune response [89]. Moreover, it is a complex procedure with a potential risk of false results influenced by subjective visual scoring. Therefore it frequently needs to be performed at reference laboratories and, for that reason, it consumes more time and expenses [90].

A newer, first-step ELISA, the C6 antibody test, uses a peptide from the constant region of a *B. burgdorferi* protein called Vmp-like sequence lipoprotein E (VlsE) [91]. Anti-VlsE antibodies develop early therefore C6 test provides comparable to higher sensitivity than a standard two-tier test [84, 92]. As it is less time-consuming than a standard two-tier test, it may be helpful in early decision whether to consider treatment [92]. Nevertheless, C6 seroprevalence in healthy blood donors tends to be high in endemic areas [93].

Branda et al. postulated an alternative diagnostic algorithm, in which WB is replaced by C6 ELISA as the second step in modified two-tier test (MTTT) [94]. As evaluated by Molins et al., it offered a simplified procedure as it was free of WB limitations. MTTT had enhanced sensitivity in diagnosing LD and was equivalent or significantly better than the standard algorithm [84]. Therefore this approach should be further investigated.

## Treatment

According to current recommendations (B/III level) from the Infectious Diseases Society of America (IDSA), children with LA should be treated initially with a 28-day course of oral antibiotics: doxycycline 4 mg/kg per day in 2 divided doses (maximum of 100 mg per dose), cefuroxime axetil 30 mg/kg per day in 2 divided doses (maximum of 500 mg per dose) or amoxicillin 50 mg/kg per day in 3 divided doses (maximum of 500 mg per dose) [35]. Doxycycline is typically preferred because of its activity against *B. burgdorferi sensu lato*. However, doxycycline is not recommended for children <8 years of age due to its contribution to discoloration of teeth [95].

In patients who are still symptomatic after the recommended treatment, another 4-week course of oral antibiotics or a 2–4-week course of intravenous ceftriaxone should be administered [35]. Nevertheless, there is no benefit in further prolongation of antibiotic therapy. In a study performed by Berende et al. there was no significant difference of health-related quality of life between groups of patients who received 2 weeks of intravenous ceftriaxone and then continued treatment with a 12-week course of oral doxycycline, clarithromycin plus hydroxychloroquine or placebo [96].

As even antibiotic-refractory cases of LA were shown by Steere et al. to eventually improve [31], further treatment is questionable. In persistent cases, symptomatic therapies with non-steroidal anti-inflammatory drugs (NSAIDs), disease-modifying antirheumatic drugs (DMARDs), or intra-articular corticosteroids are recommended in the United States according to the IDSA [35]. Oral or intra-articular corticosteroids are contraindicated until antibiotic treatment is completed since these drugs permit greater growth of spirochetes [97], and because intra-articular steroid injections were reported to prolong duration of LA [3]. DMARDs are typically prescribed for only 6–12 months rather than indefinitely as in the treatment of patients with JIA. Arthroscopic synovectomy may be an option when pharmacotherapy is eventually ineffective [98].

Spirochetal infection provides plethora of potential therapeutic targets for new designer drugs [99]. These include, among others: ClpP protease which is upregulated by spirochetal persister cells [100] and HtrA protease which plays a role in pathogenicity of *B. burgdorferi* [101].

## Conclusions

The clinical picture of LA resembles a classic autoimmune arthritis, similar to other rheumatoid disease, with immunomodulation and tissue damage. The active infection requiring antibiotic treatment is a factor that constitutes the essential difference. As a single-centre experience, the authors recommend ELISA screening in all patients suspected with JIA in regions endemic for LD. As WB confirmation may be time-consuming, simultaneous two-tier testing is advisable when patient’s history suggests a possibility of potential tick bite.

New diagnostic approaches should be elaborated on to evaluate their relevance. The assessment of the C6 antibody test in children is particularly essential for the future progress in diagnostic process and therapy.

Development of consistent worldwide guidelines would simplify diagnostic process in everyday pediatric practice.

## Abbreviations

apoB-100: apolipoprotein B-100
*B. burgdorferi*: *Borrelia burgdorferi*
CDC: The centers for disease control and prevention
DMARDs: Disease modifying antirheumatic drugs
ECGF: Endothelial cell growth factor
ELISA: Enzyme-linked immunosorbent assay
EM: Erythema migrans
HLA-DR: Human leukocyte antigen-D-related
IDSA: Infectious diseases society of America
IFN-γ: Interferon gamma
IL: Interleukin
JIA: Juvenile idiopathic arthritis
LA: Lyme arthritis
LD: Lyme disease
MMP-10: metalloproteinase 10
MTTT: Modified two-tier test
NapA: Neutrophil activating protein A
NK: Natural killer
NSAIDs: Non-steroidal anti-inflammatory drugs
OspC: Outer surface protein C
PCR: Polymerase chain reaction
RF: Rheumatoid factor
RST: Ribosomal spacer type
Th: T helper
TNF-α: Tumor necrosis factor alpha
VlsE: Vmp-like sequence lipoprotein E
WB: Western immunoblot test
